# Relationship Between Musculoskeletal Disorders and Productivity Loss Among Hospital Nurses: An Analytical Cross‐Sectional Study With Secondary Data Analysis

**DOI:** 10.1111/jnu.70020

**Published:** 2025-05-21

**Authors:** Minkyung Kang, Inah Kim, Chang Park, Ari Min

**Affiliations:** ^1^ College of Nursing, Research Institute of Nursing Science Ajou University Suwon South Korea; ^2^ College of Nursing Keimyung University Daegu South Korea; ^3^ College of Nursing University of Illinois Chicago Illinois USA; ^4^ Department of Nursing Chung‐Ang University Seoul South Korea

**Keywords:** absenteeism, musculoskeletal disorder, nurses, presenteeism, productivity, work limitation

## Abstract

**Aim:**

To identify the prevalence of musculoskeletal disorders among hospital nurses and explore their effects on productivity loss.

**Design:**

An analytical cross‐sectional study with secondary data analysis was conducted.

**Methods:**

Data were collected via an online survey of 607 registered nurses working in general and tertiary hospitals in South Korea. Multivariate logistic regression analysis was performed to examine the association between musculoskeletal disorders and four productivity loss indicators: absenteeism, presenteeism, perceived productivity loss, and work limitations.

**Results:**

Musculoskeletal disorders were highly prevalent among hospital nurses, with 83.9% of participants reporting musculoskeletal disorder symptoms in the past week. Lower back complaints had the highest prevalence. Nurses with musculoskeletal disorders were 3.74 times more likely to experience presenteeism than those without musculoskeletal disorders. They were also 3.00 times more likely to report perceived productivity loss and 2.24 times more likely to experience work limitations. However, no significant relationship was observed between musculoskeletal disorders and absenteeism.

**Conclusion:**

Musculoskeletal disorders contribute to presenteeism, productivity loss, and work limitations among hospital nurses. Targeted interventions for preventing and managing musculoskeletal disorders are essential to mitigate productivity losses and improve nurses' health and job performance. Strategies such as ergonomic workplace modifications, early detection, and effective management of musculoskeletal disorders can help maintain nurses' productivity and well‐being.

**Clinical Relevance:**

Addressing musculoskeletal disorders is critical for enhancing nurse productivity and for ensuring the delivery of high‐quality patient care. Healthcare organizations can safeguard nurses' health and patient outcomes by reducing presenteeism and work limitations.

## 1 Introduction

Musculoskeletal disorders (MSDs) are a prevalent occupational health issue among nurses worldwide. Hospital nurses are particularly vulnerable to MSDs because of the physically demanding nature of their work (Ou et al. 2021). These disorders encompass various conditions involving muscles, bones, and joints, often resulting from repetitive tasks, heavy lifting, and sustained awkward postures inherent to nursing duties (Aleid et al. 2021; Kim and Jeong 2024). A meta‐analysis reported that the annual prevalence of work‐related MSDs among nurses was alarmingly high, with up to 77.2% of nurses experiencing such conditions, particularly in the lower back, neck, and shoulders (Sun et al. 2023). This high prevalence is a global phenomenon affecting nurses across different regions. For instance, in Saudi Arabia, 63.8% of nurses reported discomfort in their lower back over the past year, illustrating the widespread nature of this issue (Tariah et al. 2020). Similarly, studies conducted in China (Wang et al. 2024), Brazil (Santos et al. 2018), and the United Kingdom (Fiorini et al. 2020) have documented significant rates of MSDs among nurses, underscoring the universal nature of this occupational hazard. These trends are mirrored in South Korea, where high patient‐to‐nurse ratios and extended working hours contribute significantly to the incidence of MSDs (Hwang et al. 2024; Kim and Jeong 2024). Despite this, limited research has specifically addressed the impact of MSDs on South Korean hospital nurses, leaving a critical gap in the literature.

The impact of MSDs extends beyond the health of individual nurses, significantly affecting productivity and economic outcomes in healthcare settings. Productivity loss can be assessed in various forms, such as absenteeism (missed workdays owing to illness), presenteeism (working despite illness), perceived productivity loss (self‐reported decline in work efficiency), and work limitations (difficulty performing essential tasks; Santos et al. 2018; Sousa et al. 2023; Zhang et al. 2023). Among these, absenteeism can lead to immediate staffing shortages, disrupting hospital operations and increasing workload burdens on remaining staff (Tariah et al. 2020). However, presenteeism may have even more severe consequences, as nurses who continue working despite MSD‐related discomfort often experience reduced efficiency, higher fatigue, and an increased risk of errors, ultimately compromising patient safety (da Silva Santos et al. 2023; Santos et al. 2018).

Beyond its immediate effects, presenteeism also contributes to long‐term productivity declines within healthcare systems. One study found that nursing workers with MSD symptoms were 2.2–6.5 times more likely to experience presenteeism, leading to an estimated 8.8% reduction in overall productivity (da Silva Santos et al. 2023). Moreover, presenteeism contributes to chronic fatigue, burnout, and declining work efficiency over time (Li et al. 2021; Shan et al. 2020). The economic burden of MSD‐related presenteeism is substantial. For instance, Japan incurs an estimated $27 billion annually owing to presenteeism linked to MSDs (Yoshimoto et al. 2020); whereas in South Korea, the cost of productivity loss from sickness presenteeism among nurses amounts to approximately USD 382.11 million per year (Kang et al. 2025). These findings highlight the urgent need for workplace interventions aimed at mitigating the negative effects of both absenteeism and presenteeism, ensuring sustainable workforce efficiency and patient safety.

In addition to absenteeism and presenteeism, MSDs also impact nurses' perceived productivity and work limitations, further contributing to workforce inefficiencies. Nurses experiencing MSD symptoms often report a subjective decline in their ability to perform job tasks effectively, which can translate into measurable work limitations (Tariah et al. 2020). MSDs are frequently accompanied by depressive symptoms (Zhang et al. 2020), which may exacerbate perceived productivity loss and increase the likelihood of work limitations. When nurses feel physically or mentally strained, their capacity to perform essential duties diminishes, leading to task redistribution among colleagues. This increased workload for other staff members can heighten stress, disrupt team dynamics, and ultimately reduce overall team efficiency. The cumulative effects of MSDs thus extend beyond the individual nurse, creating organizational challenges that impact patient care quality and healthcare system sustainability.

Despite the global recognition of these issues, research specifically addressing South Korean hospital nurses remains scarce. Further, comprehensive investigations into the direct relationships between MSDs and various dimensions of productivity loss, such as absenteeism, presenteeism, perceived productivity loss, and work limitations, are limited. Understanding the relationship between MSDs and productivity loss is essential to develop effective interventions to support nurses' health and enhance workplace productivity. Therefore, this study aimed to address these gaps by examining the prevalence of MSDs among hospital nurses in South Korea and investigating their impact on productivity loss, specifically focusing on absenteeism, presenteeism, perceived productivity loss, and work limitations. Given the physically demanding nature of nursing, we expect MSDs to be highly prevalent among South Korean hospital nurses, comparable to or exceeding international reports. Additionally, we hypothesized that nurses with MSDs will be more likely to experience greater productivity loss, reflected in high rates of absenteeism and presenteeism, as well as high perceived productivity loss and work limitations. The findings offer valuable insights for policymakers and healthcare administrators to develop targeted interventions to mitigate the adverse effects of MSDs and optimize patient care outcomes.

## 2 Materials and Methods

### 2.1 Study Design, Setting, and Sample

This study employed an analytical cross‐sectional study design using secondary data analysis. Data were obtained from a study on nurses' presenteeism (Kang et al. 2025; Seo et al. 2024). Participants were recruited using convenience sampling through the mobile application *MyDuty*, widely used by approximately 90% of shift‐working nurses in South Korea for managing shift schedules. An invitation to participate was posted within the application, and eligible nurses voluntarily completed an online survey administered via Google Forms. The inclusion criteria were: (a) registered nurses working in general or tertiary hospitals (≥ 300 beds), and (b) those directly involved in patient care. Newly hired nurses in training or those not currently working in a hospital setting were excluded.

As this study involved secondary data analysis, we conducted a post hoc power analysis using G*Power software version 3.1.9.7 to confirm that the available sample size was sufficient. With an assumed moderate effect size (odds ratio = 2.0), an alpha of 0.05, and power of 0.95 for logistic regression analysis, the required minimum sample size was calculated to be 455. Owing to the characteristics of the Google Forms platform, data were saved only for fully completed submissions; thus, the exact number of nurses initially accessing or partially completing the survey was unavailable, making the calculation of the initial response rate impossible. Ultimately, among the 608 nurses who completed and submitted the survey, data from 607 respondents were included in the final analysis after excluding one invalid response owing to data inconsistencies, resulting in a completion rate of 99.8%.

### 2.2 Data Collection

Data were collected via an online survey using Google Forms, administered to participants' mobile devices on January 3–4, 2023. Nurses who viewed the study advertisement through the mobile application voluntarily completed screening questions to determine eligibility. Only nurses meeting the inclusion criteria proceeded to the full survey after providing informed consent. Completing the survey took approximately 10–15 min, and responses were recorded only upon submission of the completed questionnaire.

To ensure participant confidentiality, each respondent was assigned a unique identification number, thereby anonymizing responses by removing all personal identifiers. Collected survey data and informed consent forms were encrypted, password‐protected, and stored separately on the principal investigator's personal computer, accessible exclusively by authorized research team members. In compliance with Article 15 (2) of the Bioethics and Safety Act of South Korea, all research‐related documents and data are securely retained for 3 years following study completion. Access to the stored data was strictly limited to the research team and used solely for research purposes.

### 2.3 Measures

#### 2.3.1 General Characteristics

Participants' general characteristics included age (in years), sex, work experience as a registered nurse (in years), working hours during the previous week (in hours), overtime hours during the previous week (in hours), and breaks during work (in min; Kang et al. 2025; Seo et al. 2024). Working hours were categorized into two groups—40 h or less and more than 40 h—based on the Labor Standards Act in South Korea, which states that an employee's weekly working hours should not exceed 40 h without an agreement between the employer and employee (National Assembly of the Republic of Korea 2021). For overtime hours and rest breaks, participants were first asked whether they had worked overtime or had taken a rest break, and only those who answered “yes” provided the total overtime hours and rest break durations in minutes.

#### 2.3.2 Productivity Loss

Table 1 summarizes the definitions and cutoff points used in the dichotomous classification of the productivity loss indicators. The variables include absenteeism (sick leave), presenteeism, perceived productivity loss, and work limitation, each divided into “normal productivity” and “productivity loss.”

**TABLE 1:** Summary of definition and cutoff points used in dichotomy categories of productivity loss indicators.

Variables	Definition	
	Normal productivity	Productivity loss
Absenteeism	Did not take any sick leave from work in the past month	Took sick leave from work in the past month
Presenteeism	Did not have health problems or did not work while sick in the past month	Had health problems in the past month and yet went for work
Perceived productivity loss	Perceived productivity loss score during presenteeism < median	Perceived productivity loss score during presenteeism ≥ median
Work limitation	WLQ productivity loss score ≤ healthy benchmark sample (who did not experience MSD) mean WLQ productivity loss score value	WLQ productivity loss score > healthy benchmark sample (who did not experience MSD) mean WLQ productivity loss score value

Abbreviations: MSD, musculoskeletal disorders; WLQ, work limitations questionnaire.

Absenteeism and presenteeism were assessed using items from the Korean Working Conditions Survey, which were adapted from the European Working Conditions Survey through a rigorous translation and cultural adaptation process to ensure validity in the Korean context (Cho 2023). Participants were asked if they had been absent from work owing to illness in the past 4 weeks to measure absenteeism, and whether they had worked while sick during the same period to assess presenteeism (Kang et al. 2025). Participants who answered “yes” to these questions were considered to have experienced either absenteeism or presenteeism. Those who did not take sick leave were classified as having normal productivity, whereas those who took sick leave were categorized as experiencing productivity loss. For presenteeism, participants who had no health problems or did not work while sick were categorized as having normal productivity. Conversely, those who worked while experiencing health issues were classified as experiencing productivity loss.

Perceived productivity loss during presenteeism was assessed using a single item from the Korean version of the Work Productivity and Activity Impairment questionnaire (WPAI), originally developed by Reilly, Zbrozek, and Dukes (1993). The WPAI has undergone rigorous translation and cultural adaptation processes to ensure its validity and reliability across various linguistic and cultural contexts. Notably, it has been translated into more than 140 languages through a harmonization process involving multiple independent translations and back‐translations (Reilly Associates, n.d.). The specific item used in this study asks, “During the past 7 days, how much did health problems affect your productivity while you were working?” Participants rated the extent to which their health problems affected their productivity over the past 7 days on a scale of 0 (no effect) to 10 (completely prevented from working). Participants who had not experienced presenteeism were assigned a score of 0. Perceived productivity loss was then categorized by the median score; those scoring below the median were classified as having normal productivity, and those scoring at or above the median were classified as having productivity loss.

Work limitations were assessed using the Korean version of the Work Limitations Questionnaire (WLQ), originally developed by Lerner et al. (2001). The WLQ comprises 25 items evaluating the extent to which health conditions interfere with job performance. For this study, we used the Korean version of the WLQ provided by ePROVIDE (Mapi Research Trust 2022). The items were grouped into four subscales: time management demands (five items), physical demands (six items), mental‐interpersonal demands (nine items), and output demands (five items). Participants rated their limitations over the past 2 weeks on a five‐point scale, and the WLQ productivity loss score was calculated as the weighted sum of these subscales (Seo et al. 2024). The scores ranged from 0% (no limitation) to 24.9% (maximal limitation). The WLQ has shown high construct validity and reliability in the development study (Cronbach's alphas between 0.88 and 0.91; Lerner et al. 2001). The Cronbach's alphas for this study were 0.85 (time management demands), 0.83 (physical demands), 0.90 (mental‐interpersonal demands), and 0.90 (output demands). Work limitations were dichotomized using a healthy benchmark sample (without MSDs), with participants scoring below or equal to the mean classified as having normal productivity and those exceeding this benchmark categorized as experiencing productivity loss.

#### 2.3.3 MSDs

MSDs were assessed using the standardized Nordic Musculoskeletal Questionnaire (NMQ), developed from a project supported by the Nordic Council of Ministers (Kuorinka et al. 1987). The questionnaire has shown acceptable reliability with the test–retest method in development studies (Kuorinka et al. 1987), has been translated and validated in many countries, and is widely used by nursing personnel (Dawson et al. 2009). For this study, we used the Korean version of the NMQ, which has been translated and culturally adapted to ensure measurement validity within the Korean population (Choi et al. 2008). The Korean NMQ has undergone validation through clinical examinations and has been demonstrated to have high sensitivity (73.9%) and specificity (68.0%) for screening and surveillance of work‐related MSDs in various occupational groups (Choi et al. 2008). It comprises forced choices (yes/no) for nine body parts: neck, shoulders, upper back, elbows, lower back, wrists/hands, hips/thighs, knees, and ankles/ft. Participants were asked if they had any musculoskeletal problems (ache, pain, or discomfort) in any body part in the last 12 months (chronic) and the last 7 days (acute). In this study, total MSDs were defined as participants experiencing MSDs in at least one of the nine body parts during either period.

### 2.4 Data Analysis

All statistical analyses were conducted using complete data, as the Google Forms survey required participants to answer all questions fully before submission, resulting in no missing data. Descriptive statistics, including means, standard deviations, medians, interquartile ranges, frequencies, and percentages, were used to describe demographics and study variables. The total MSDs for the past 12 months and 7 days were quantified for each participant who complained of pain in at least one body part. A chi‐square test was used to investigate the differences between MSDs and productivity loss. Finally, multivariate logistic regression analysis was performed to examine the association between MSDs and four productivity loss indicators: absenteeism, presenteeism, perceived productivity loss, and work limitations. Adjusted odds ratios (AORs) and 95% confidence intervals (CIs) were estimated to control for potential confounders, such as age, sex, work experience, working hours, overtime hours, and rest breaks. Data analyses were performed using STATA (version 18.0, College Station, TX, USA). We estimated AORs and 95% CIs. We set significance at *p* < 0.05.

### 2.5 Ethical Consideration

Secondary data analysis was conducted using anonymized data following approval from the Chung‐Ang University Institutional Review Board (no. 1041078‐20240129‐HR‐014). The data used in this analysis were originally collected after obtaining ethical approval from the Chung‐Ang University Institutional Review Board (no. 1041078‐202211‐HR‐255; Seo et al. 2024). All participants received detailed information about the study objectives, procedures, data confidentiality, and voluntary participation on the first page of the online survey. Those who agreed to participate provided consent by signing an online form before beginning the survey. Participants were informed that they could withdraw at any time without penalty, and only data from the completed surveys were stored in the database.

## 3 Results

### 3.1 Participants' Characteristics

Participants' demographic and occupational characteristics and their comparison by MSD status are summarized in Table 2 and elsewhere (Kang et al. 2025). Most were women (93.6%), aged younger than 30 years (mean = 30.37 years, standard deviation [SD] = 5.49), and reported working overtime during the past week (mean = 4.15 h, SD = 4.97). Additionally, 87.6% of participants had a rest break (including meal break) during work, with an average duration of 19.99 min (SD = 16.73). Chi‐square analyses revealed that MSD prevalence significantly differed by sex and overtime work status; female nurses and those working overtime were more likely to experience MSDs (*p* = 0.003 and *p* < 0.001, respectively). MSD prevalence did not significantly differ by age, work experience, weekly working hours, or rest breaks (all *p* > 0.05).

**TABLE 2:** Comparison of musculoskeletal disorders by participants' characteristics (*N* = 607).

Characteristics	Category	Total	MSD (*n* = 509)	Without MSD (*n* = 98)	*χ* ^2^	*p*
		*n* (%)				
Age (years)	< 30	302 (49.8)	250 (82.8)	52 (17.2)	0.535	0.765
	30–39	263 (43.3)	223 (84.8)	40 (15.2)		
	≥ 40	42 (6.9)	36 (85.7)	6 (14.3)		
Sex	Female	568 (93.6)	483 (85.0)	85 (15.0)	9.095	0.003
	Male	39 (6.4)	26 (66.7)	13 (33.3)		
Work experience as a nurse (years)	≤ 1	86 (14.2)	75 (87.2)	11 (12.8)	1.921	0.589
	2–5	236 (38.9)	197 (83.5)	39 (16.5)		
	6–10	196 (32.3)	160 (81.6)	36 (18.4)		
	> 10	89 (14.6)	77 (86.5)	12 (13.5)		
Working hours (h, per week)	≤ 40	197 (32.5)	163 (82.7)	34 (17.3)	0.267	0.605
	> 40	410 (67.5)	346 (84.4)	64 (15.6)		
Overtime hours (h, per week)	Yes	503 (82.9)	436 (86.7)	67 (13.3)	17.305	< 0.001
	No	104 (17.1)	73 (70.2)	31 (29.8)		
Rest break during work^a^ (min)	Yes	532 (87.6)	443 (83.3)	89 (16.7)	1.086	0.297
	No	75 (12.4)	66 (88.0)	9 (12.0)		

Abbreviations: MSD, musculoskeletal disorder; SD, standard deviation.

^a^ Including meal break.

### 3.2 Prevalence of MSD

Table 3 presents detailed prevalence rates of MSD symptoms among participants for the past 12 months and past 7 days. Over the past 12 months, the vast majority (94.6%) reported experiencing MSD symptoms in at least one body part, with the lower back, neck, and shoulders being the most frequently affected areas. The prevalence rates for MSD symptoms reported in the past 7 days were slightly lower (83.9%), yet the lower back, shoulders, and neck remained the most commonly affected regions. Less commonly affected areas included wrists/hands, knees, ankles/ft, upper back, hips/thighs, and elbows.

**TABLE 3:** Prevalence of musculoskeletal disorders among hospital nurses (*N* = 607).

Body parts	Prevalence, *n* (%)	
	12 months	7 days
Neck	373 (61.5)	241 (39.7)
Shoulders	366 (60.3)	247 (40.7)
Elbows	38 (6.3)	27 (4.5)
Wrists/hands	257 (42.3)	172 (28.3)
Upper back	132 (21.8)	64 (10.5)
Lower back	413 (68.0)	280 (46.1)
Hips/thighs	86 (14.2)	47 (7.7)
Knees	206 (33.9)	113 (18.6)
Ankles/ft	192 (31.6)	107 (17.6)
Any body parts (total MSDs)	574 (94.6)	509 (83.9)

Abbreviation: MSDs, musculoskeletal disorders.

### 3.3 Productivity Loss

Approximately 10.9% of participants (*n* = 66) reported taking sick leave from work, while 63.9% (*n* = 388) reported working despite having health problems (i.e., presenteeism) in the past month. The median perceived productivity loss score during presenteeism was 4 (interquartile range = 0–7), and this value was used as a cutoff point to differentiate between the normal productivity and productivity loss groups based on the perceived productivity loss score. The average WLQ productivity loss score was 8.84 (SD = 4.48). Among the nurses who reported MSDs in the past week, the mean WLQ productivity loss score was 9.16 (SD = 4.38). In contrast, nurses without MSDs in the past week had a mean WLQ productivity loss score of 7.11 (SD = 4.63), which was used as the cutoff point to distinguish between the normal productivity and productivity loss groups for the work limitation variable.

### 3.4 Associations Between MSDs and Productivity Loss

Associations between MSDs and productivity loss indicators are summarized in Table 4. MSDs experienced in the past week showed significant associations with presenteeism, perceived productivity loss, and work limitations, but not with absenteeism. Specifically, nurses with MSDs were significantly more likely to experience presenteeism (*p* < 0.001), perceived productivity loss (*p* < 0.001), and work limitations (*p* < 0.001). Interestingly, despite the clear associations observed for these indicators, MSDs did not significantly increase absenteeism (*p* = 0.195). Multivariate logistic regression analysis confirmed these associations, showing that nurses with MSDs had significantly increased odds of presenteeism (AOR = 3.74), perceived productivity loss (AOR = 3.00), and work limitations (AOR = 2.24; Table 5).

**TABLE 4:** Associations between musculoskeletal disorders (past week) and productivity loss indicators.

Variables		Productivity		*χ* ^2^	*p*
		Normal	Loss		
Absenteeism	MSD	450	59	1.68	0.195
	Without MSD	91	7		
Presenteeism	MSD	157	352	37.45	< 0.001
	Without MSD	62	36		
Perceived productivity loss^a^	MSD	210	299	26.01	< 0.001
	Without MSD	68	30		
Work limitation	MSD	171	338	16.29	< 0.001
	Without MSD	54	44		

Abbreviation: MSD, musculoskeletal disorder.

^a^ The perceived productivity loss score of participants who did not experience presenteeism is 0.

**TABLE 5:** Multivariate logistic regression analysis on effects of musculoskeletal disorders on productivity loss indicators.

Variables	Odds ratio	95% confidence intervals		*p*
		Upper	Lower	
Absenteeism				
MSD	1.71	0.75	3.92	0.200
Without MSD	1.00			
Presenteeism				
MSD	3.74	2.34	5.96	< 0.001
Without MSD	1.00			
Perceived productivity loss				
MSD	3.00	1.86	4.86	< 0.001
Without MSD	1.00			
Work limitation				
MSD	2.24	1.42	3.53	0.001
Without MSD	1.00			

*Note:* The analysis was adjusted for age, sex, work experience, working hours, overtime hours, and rest breaks.

Abbreviation: MSD, musculoskeletal disorder.

## 4 Discussion

This study investigated the prevalence of MSDs among hospital nurses in South Korea and their impact on productivity loss, specifically focusing on absenteeism, presenteeism, perceived productivity loss, and work limitations. The findings demonstrated that MSDs are highly prevalent among hospital nurses, with 94.6% of participants reporting MSDs in at least one body part in the past 12 months and 83.9% in the past week. The lower back, shoulders, and neck were the most affected areas. Nurses with MSDs were significantly more likely to experience presenteeism, perceived productivity loss, and work limitations; however, no significant association was observed with absenteeism.

### 4.1 The Burden of MSDs Among Hospital Nurses

The high prevalence of MSDs found in this study—94.6% over the past 12 months and 83.9% over the past week—aligns with global trends, but is particularly alarming when compared to similar studies in other countries. For instance, a meta‐analysis by Sun et al. (2023) reported an average global MSD prevalence of 77.2% among nurses, with lower back, neck, and shoulder complaints being the most common. Our findings showed comparatively higher rates, particularly in the lower back and shoulders, suggesting that South Korean hospital nurses may face additional risk factors related to their work environment.

In comparison to other countries, such as Saudi Arabia, where 63.8% of nurses reported lower back discomfort (Tariah et al. 2020), and China, where nurses also reported high rates of MSDs (Wang et al. 2024), the rates in South Korea were notably higher. This could be attributed to specific working conditions in Korean hospitals, such as high patient‐to‐nurse ratios and long working hours, which are known risk factors for MSDs (Kim and Jung 2016; Kim et al. 2014; Lee et al. 2018). These conditions likely exacerbate the physical strain on nurses, making them more vulnerable to musculoskeletal issues. Moreover, the prevalence of MSDs in this study surpasses that of nurses in European countries, such as the United Kingdom, where the prevalence of MSDs was lower (Fiorini et al. 2020). This disparity may reflect differences in healthcare systems, working hours, and availability of ergonomic interventions. In many European countries, stricter labor regulations, better staff‐to‐patient ratios, and more advanced interventions may help mitigate the physical burden on nurses.

The heightened prevalence of MSDs in South Korea underscores the need for tailored interventions. The significantly higher prevalence compared to the global averages suggests that South Korean hospital nurses may face unique or amplified risk factors requiring urgent attention. A systematic review of interventions for preventing musculoskeletal injuries in nurses reported that the most effective strategies included handling device training and ergonomics education to prevent work‐related MSDs (Sousa et al. 2023). Occupational health initiatives, such as providing assistive devices, implementing ergonomic training programs, and introducing health‐monitoring systems may be more crucial in South Korea than in other regions with lower MSD rates. Further, future interventions should be tailored to the specific types of MSDs most common in South Korean hospital nurses, ensuring the implementation of effective strategies.

### 4.2 MSDs and Their Impact on Productivity Loss

One of the critical findings was the robust association between MSDs and productivity loss in the form of presenteeism, perceived productivity loss, and work limitations. This mirrors the findings of other studies, but the magnitude of the impact observed in this study is especially concerning. Nurses with MSDs were 3.74 times more likely to experience presenteeism and 3.00 times more likely to report perceived productivity loss than their counterparts without MSDs. These results align with the research by da Silva Santos et al. (2023), which highlights a significant relationship between musculoskeletal symptoms and presenteeism (OR = 1.3–6.5) as well as an overall productivity loss of 8.8% among nursing workers in Brazil. The relatively high odds ratios observed in this study suggest that South Korean nurses may experience more pronounced effects of MSDs on their ability to perform at work compared to findings from other regions.

The high rate of presenteeism observed in this study (63.9%) is particularly concerning as presenteeism can have a more detrimental effect on productivity than absenteeism (Miraglia and Johns 2016). When nurses do not operate at full capacity owing to pain, their ability to focus, make critical decisions, and provide optimal care is compromised. This can lead to medical errors, reduced patient satisfaction, and a decline in the overall quality of care provided by healthcare institutions (Rainbow et al. 2020). Further, presenteeism often proves more detrimental than absenteeism, as its impacts remain less visible but are more pervasive (Nowak et al. 2023).

An important and noteworthy finding of this study was that MSDs showed significant associations with presenteeism, perceived productivity loss, and work limitations, but interestingly, no significant relationship with absenteeism was observed. This finding suggests nurses tend to continue working despite considerable musculoskeletal discomfort, which underscores the critical issue of presenteeism. Possible explanations for this phenomenon could include professional obligations, concerns about burdening colleagues, or workplace cultures that discourage taking leave even when experiencing health issues (Madrazo et al. 2025; Szymczak 2017). This finding highlights the necessity of workplace interventions focusing explicitly on addressing presenteeism and improving working conditions rather than relying solely on absenteeism as an indicator of nurses' health and productivity.

Work limitations associated with MSDs, in which nurses are restricted in their ability to perform essential tasks, underscore broader implications for healthcare delivery. These limitations hinder nurses' physical capacity to perform tasks, such as lifting patients, administering medication, or completing procedures efficiently. This can have a ripple effect, as incomplete or improperly executed tasks may create additional burdens for colleagues, leading to heightened stress levels and an increased risk of burnout among nursing staff. Moreover, workplace conflicts can significantly increase sickness presenteeism, negatively impacting teamwork and reducing overall productivity (Lakiša et al. 2022). Thus, the impact of MSDs on productivity should be recognized both as a personal health issue for nurses and as a systemic challenge affecting the entire healthcare delivery system.

The relationship between MSDs and productivity could also be bidirectional, as both factors influence each other. MSDs reduce productivity by impairing nurses' physical capacity and work efficiency, making it difficult to perform essential tasks such as patient handling, medication administration, and clinical procedures (Ou et al. 2021). Simultaneously, high workloads, inadequate staffing, and extended working hours contribute to MSDs, as nurses face sustained physical strain with limited opportunities for recovery (Kowalski et al. 2021). The pressure to maintain high productivity can also lead to unsafe behaviors that increase the risk of MSDs. Nurses working under strict time constraints or heavy patient loads may adopt improper manual handling techniques, skip rest breaks, or neglect proper body mechanics, further exacerbating musculoskeletal strain (Park et al. 2021). Additionally, workplace culture and staffing policies influence how nurses manage MSD symptoms while maintaining productivity, as some organizational environments discourage taking sick leave, leading to increased presenteeism and prolonged physical stress (Lee et al. 2024). This cycle underscores the necessity of organizational interventions that prioritize MSD prevention and address systemic workplace pressures that contribute to their occurrence. Future research should examine the interplay among workplace policies, staffing levels, and job demands related to MSD prevalence. Such investigations could inform comprehensive strategies aimed at simultaneously enhancing nurse well‐being and optimizing productivity while fostering a healthier work environment.

### 4.3 Implications for the Nursing Profession

These findings have profound implications for the nursing profession, particularly in South Korea's healthcare system. The high prevalence of MSDs and their associated productivity losses, such as presenteeism, perceived productivity loss, and work limitations, highlight the need for systemic changes in healthcare environments to better support the physical health of nurses. High patient‐to‐nurse ratios and the extended working hours commonly seen in South Korean hospitals likely exacerbate the risk of MSDs, contributing to higher rates of presenteeism and work limitations than those observed in other countries. These working conditions place additional strain on nurses and underscore the urgency of targeted interventions aimed at reducing these burdens.

MSDs are both a personal health issue and a systemic problem that affects the overall functioning of healthcare systems. A critical area for intervention concerns workplace policies that prioritize nurses' health and safety. This includes creating a supportive environment in which nurses feel empowered to take sick leave without fear of penalty or judgment. Many nurses engage in presenteeism because of a sense of duty or fear of burdening their colleagues. Healthcare administrators should foster a culture that emphasizes the importance of rest and recovery.

Healthcare institutions should also invest in preventive strategies such as ergonomic training programs, physical therapy, and regular health screenings for nurses. Introducing assistive technologies, such as mechanical lifts for patient transfers can reduce the physical strain on nurses, whereas flexible scheduling and adequate rest periods can help mitigate the cumulative effects of MSDs. These interventions will enhance nurses' well‐being and improve job satisfaction and retention, both of which are critical in an era of nursing shortages.

Additionally, these findings highlight the need for more comprehensive occupational health programs in nursing. While nursing education often includes training in body mechanics and injury prevention, ongoing education and resources are essential for addressing the evolving physical demands of the profession. Nurses should be regularly assessed for MSD risks and personalized interventions that can prevent the development or exacerbation of these conditions.

### 4.4 Limitations and Future Directions

This study has some limitations. First, using convenience sampling via the MyDuty mobile application may have introduced selection bias, as nonusers may differ from participants, limiting the generalizability of the findings. Second, the cross‐sectional design prevents establishing causal relationships between MSDs and productivity loss. Longitudinal studies tracking MSD progression and its impact on productivity over time are needed to better understand its chronic nature. Third, reliance on self‐reported data introduces the risk of response bias, particularly in assessing presenteeism and productivity loss. Future studies should incorporate objective measures, such as medical records or observational data, to enhance accuracy. Fourth, potential self‐selection bias should be considered, as nurses with MSD symptoms may have been more motivated to participate, possibly leading to an overestimation of MSD prevalence and its impact on productivity. Fifth, the study's focus on hospital nurses in South Korea limits the generalizability of findings to other healthcare settings and countries. Future research should explore MSD prevalence and productivity loss across different nursing specialties and settings, such as long‐term care and outpatient clinics, to develop targeted interventions. Lastly, the absence of a significant association between MSDs and absenteeism requires further examination. Nurses with MSDs may modify work tasks to reduce physical strain, or seek assistance to avoid absenteeism, while workplace norms influenced by workforce shortages may discourage taking leave. Future research should investigate how MSDs affect work adjustments and explore the role of organizational culture in absenteeism behaviors.

## 5 Conclusion

This study highlights the substantial burden of MSDs among hospital nurses in South Korea and their negative impact on productivity, particularly through presenteeism, perceived productivity loss, and work limitations. Given the high prevalence of MSDs and their association with workplace inefficiencies, addressing these issues is essential for improving both nurse well‐being and healthcare system performance. Our findings underscore the need for further research to identify effective strategies for reducing MSD‐related productivity loss. While this study establishes an association between MSDs and productivity outcomes, it does not assess the impact of specific workplace interventions. Future research should explore how workplace policies, staffing conditions, and organizational culture influence MSD management and productivity among nurses. By advancing research on MSD prevention and workplace conditions, healthcare institutions can develop evidence‐based strategies that support nurses' health, enhance productivity, and sustain high‐quality patient care.

### 5.1 Clinical Resources

National Institute for Occupational Safety and Health (NIOSH; November 2024) Ergonomics and Musculoskeletal Disorders. https://www.cdc.gov/niosh/ergonomics/.

## Conflicts of Interest

The authors declare no conflicts of interest.

## Data Availability

The data supporting the findings of this study are available from the corresponding author upon reasonable request.
